# β-Defensin 129 Attenuates Bacterial Endotoxin-Induced Inflammation and Intestinal Epithelial Cell Apoptosis

**DOI:** 10.3389/fimmu.2019.02333

**Published:** 2019-10-04

**Authors:** Kunhong Xie, Hongmei Xie, Guoqi Su, Daiwen Chen, Bing Yu, Xiangbing Mao, Zhiqing Huang, Jie Yu, Junqiu Luo, Ping Zheng, Yuheng Luo, Jun He

**Affiliations:** ^1^Institute of Animal Nutrition, Sichuan Agricultural University, Chengdu, China; ^2^Key Laboratory of Animal Disease-resistant Nutrition, Chengdu, China; ^3^Shandong Vocational Animal Science and Veterinary College, Weifang, China

**Keywords:** endotoxemia, inflammation, porcine β-defensin 129, intestinal epithelium, apoptosis

## Abstract

Defensins have attracted considerable research interest worldwide because of their potential to serve as a substitute for antibiotics. In this study, we characterized a novel porcine β-defensin (pBD129) and explored its role in alleviating bacterial endotoxin-induced inflammation and intestinal epithelium atrophy. The *pBD129* gene was cloned and expressed in *Escherichia coli*. A recombinant pBD129 protein was also purified. To explore its role in alleviating the endotoxin-induced inflammation, mice, with or without lipopolysaccharide (LPS) challenge were treated by pBD129 at different doses. The recombinant pBD129 showed significant antimicrobial activities against the *E. coli* and *Streptococcus* with a minimal inhibitory concentration (MICs) of 32 μg/mL. Hemolytic assays showed that the pBD129 had no detrimental impact on cell viabilities. Interestingly, we found that pBD129 attenuated LPS-induced inflammatory responses by decreasing serum concentrations of inflammatory cytokines, such as the IL-1β, IL-6, and TNF-α (*P* < 0.05). Moreover, pBD129 elevated the intestinal villus height (*P* < 0.05) and enhanced the expression and localization of the major tight junction-associated protein ZO-1 in LPS-challenged mice. Additionally, pDB129 at a high dose significantly decreased serum diamine oxidase (DAO) concentration (*P* < 0.05) and reduced intestinal epithelium cell apoptosis (*P* < 0.05) in LPS-challenged mice. Importantly, pBD129 elevated the expression level of Bcl-2-associated death promoter (Bcl-2), but down-regulated the expression levels of apoptosis-related genes such as the B-cell lymphoma-2-associated X protein (Bax), BH3-interacting domain death agonist (Bid), cysteinyl aspartate-specific proteinase-3 (Caspase-3), and caspase-9 in the intestinal mucosa (*P* < 0.05). These results suggested a novel function of the mammalian defensins, and the anti-bacterial and anti-inflammatory properties of pBD129 may allow it a potential substitute for conventionally used antibiotics or drugs.

## Introduction

Endotoxemia induced by bacterial endotoxins involves a series of responses, including secretion of pro-inflammatory mediators, expression of adhesion molecules, and multiple organ dysfunctions (1). Previous studies have indicated that endotoxemia usually caused destruction of tight junction integrity and intestinal epithelium apoptosis (2, 3), which subsequently led to disruption of intestinal homeostasis and damage of the intestinal barrier functions (4, 5). The intestinal epithelium barrier not only contributes to absorption of nutrients, but also contributes to preventing pathogens and toxins from the intestinal lumen from entering circulation (6, 7). Damage of the intestinal epithelium barrier resulted in exposure of submucosa to a variety of pathogens, which subsequently activated the innate immune response and produced a large number of pro-inflammatory cytokines, such as the interleukin-1β (IL-1β), interleukin-6 (IL-6), and tumor necrosis factor-α (TNF-α) (8). These pro-inflammatory cytokines not only caused elevated intestinal permeability, but also induced intestinal epithelial cell apoptosis (9).

The defensins, expressed in a variety of epithelial cells, are classified into alpha, beta, and theta forms based on the intramolecular disulfide bond patterns between six cysteines (10, 11). These proteins are a well-characterized group of small, disulphide-rich, cationic peptides that are highly diverse in their sequences and structures (12). Previous studies indicated that the β-defensins possess multidirectional biological properties, including antiviral, antibacterial, and anti-inflammatory effects (13, 14). However, evidence is accumulating to show that β-defensins can also play a role in regulating innate immunity and maintaining intestinal health. For instance, the β-defensin 2 was reported to attenuate inflammation and mucosal lesions during the pathological process of dextran sodium sulfate (DSS)-induced colitis (15). Moreover, the β-defensin 3 significantly decreased production of pro-inflammatory cytokines by macrophages upon *Porphyromonas gingivalis* lipopolysaccharide challenge (16). The porcine β-defensin 129 (pBD129), a newly isolated porcine β-defensin, was first identified in reproductive tissues and was found to be overexpressed in wild boars infected by mycobacteria (17, 18). Although numerous evidence indicates that multiple β-defensins can serve as a critical regulator for diverse biological events including immune responses (15–18), the involvement of pBD129 in regulating the inflammatory responses is just beginning to be explored.

In the present study, we explored the effect of pBD129 on inflammatory responses and intestinal epithelium barrier functions by using a mouse model. The *pBD129* gene was cloned and expressed in *Escherichia coli*, and a recombinant pBD129 protein was purified and characterized *in vitro*. To explore its role in regulating the endotoxin-induced inflammation, mice, with or without LPS challenge were treated by the recombinant pBD129 at different doses. Our study suggests a novel function of the mammalian defensins, and will assist in rational target selection, alleviating the endotoxemia-induced inflammation and damage of the intestinal epithelium barriers.

## Materials and Methods

### Synthesis, Expression, and Purification of PBD129

The porcine β-defensin 129 gene (GenBank accession No. NM_001129975.1) was synthesized and cloned into the Sac 1/Hind III sites of pET32a(+) by Tsingke Biological Technology Co., Ltd. (Chengdu, China). The resulting plasmid [pET32a(+)-pBD129] was transformed into *E. coli* BL21(DE3). Cultivation of the *E. coli* BL21(DE3) was performed at 37°C in LB medium supplemented with ampicillin (100 μg mL^−1^) at 200 rpm. After incubation to mid-log growth (OD600 of 1.0), 1 mM isopropyl-l-thiogalactopyranoside (IPTG) were added to induce the expression of pBD129 protein. Cells were harvested by centrifugation at 8,000 × g for 20 min at 4°C, and lysed by sonication in ice-water bath after suspending in Binding buffer (20 mM Tris-HCI, 0.5 M NaCl, 10 mM imidazole, pH 7.9). The supernatant of the cell lysate resulting from centrifugation at 8,000 × g for 30 min was applied to a Ni-NTA column (Shenggong, Shanghai). After washing to baseline absorbance with Binding buffer, the column was washed with Elution Buffer (20 mM Tris-HCI, 0.5 M NaCl, 500 mM imidazole, pH 7.9) at a flow rate of 1 mL/min. The fractions were collected and applied to 12% SDS-PAGE. The protein concentration was determined by the BCA assay (Beyotime, Shanghai, China). After dialyzing with sterile saline solution (0.09% [wt/vol] NaCl in distilled water), the purified protein pBD129 was stored at −80°C for further use.

### Mass Spectrometry Analysis

The expressed protein band was excised from gel for LC-MS/MS mass spectrometry analysis. Briefly, after the gel plug was digested with trypsin, 10 μL of the peptide mixture was separated at a flow rate of 400 nL/min on a C18-reversed phase column. A prominent nano 2D chromatography system (Shimadzu Corp., Kyoto, Japan) was attached to the mass spectrometer micrOTOF-QII (Bruker Corporation, Billerica, MA, USA). The data was collected using Bruker Daltonics micrOTOF control software 3.2 (Bruker Corporation) with the conditions 50–2,200 m/z scan range, 1,500 V capillary voltages, and 150°C drying argon gas temperature. Finally, the selected peptide masses were analyzed using Data Analysis software 4.1 (Bruker Corporation) and searched using the Mascot search engine version 2.3.01.

### Assays of the Antibacterial and Hemolytic Activities

Three Gram-positive species (*Streptococcus dysgalactiae* ATCC 12394, *Staphylococcus aureus* CICC23656, and *Bacillus subtilis*), three Gram-negative bacterial species (*E. coli* DH5α, *E. coli* K88^+^, and *Salmonella typhimurium* CICC14028), and *Pichia pastoris* X33 were used for the measurement of the antibacterial activity. The minimum inhibitory concentration (MIC) was determined by the method as previously described (19). The bacteria were grown overnight at 37°C; the culture was then diluted using medium to a concentration of 1 × 10^5^ CFU/mL and seeded into a 96-well plate at a density of 100 μL/well. Recombinant pBD129 was serially diluted from 512 μg/mL by a factor of 2, and 100 μL/well was added to the 96-well plate. The same volume peptide solutions (100 μL) without bacteria were used as negative controls. The reaction system was incubated at 37°C for 24 h. The OD600 nm was measured to calculate the MIC. The experiments were done in triplicates on the same plate. Moreover, hemolytic activity measurements were performed according to a previous study (20). Briefly, 10 mL whole porcine blood was centrifuged at 1,500 × g for 10 min at room temperature. The porcine blood cells were washed three times with PBS buffer (150 mM NaCl; 10 mM Na2HPO4/NaH2PO4, pH 7.4) and resuspended in PBS buffer (in a 25-fold diluted concentration of erythrocytes compared to blood). Subsequently, 150 μL aliquots were added to 150 μL peptide solutions (final concentration 0–256 μg/mL pBD-129) in polypropylene 96-well microtiter plates, and the mixture was incubated for 1 h at 37°C. After incubation, the plate was centrifuged for 5 min at 1,500 × g and 150 μL supernatant of each well was transferred to a new 96-well plate. Extinction was measured at 450 nm with UV-1100 spectrophotometer (ShangHai, China) and the percentage hemolysis was calculated by comparison with the control samples containing no peptide or 1% Triton X-100.

### Animal Trial

The animal trial was approved by the Animal Welfare Committee of Sichuan Agricultural University (No. 20180718). Sixty male ICR mice (4 weeks old) were purchased from Chengdu Da Shuo laboratory animal Co., Ltd. (Chengdu, China), and used for a 3 × 2 factor design (*n* = 10). The mice were intraperitoneally injected by three doses of pBD129 (0, 4, and 8 mg/kg), and challenged by sterile saline or LPS. All animals were individually housed at 22 ± 2°C with a cycle of 12 h light/12 h dark, and free access to food and water. The injections of pBD129 were carried out for 6 days (once a day) via 1 ml insulin syringe (Braun, Melsungen, Germany). At 7 d, mice were either challenged (intraperitoneal injection) by sterile saline or LPS (*Escherichia coli* O55:B5; Sigma-Aldrich, SL, USA) at a dose of 10 mg/kg. Five hours after challenge, the mice were anesthetized via 20-s exposure to carbon dioxide and subjected to cardiac blood sampling. Duodenum, jejunum, and ileum samples were taken immediately after dislocation of the neck. A portion of the sample was fixed in formaldehyde solution for morphological observation and the other portion was rapidly frozen in liquid nitrogen and stored at −80°C until analysis. Blood samples were centrifuged at 3,000 × g for 15 min at 4°C, after which the serum was separated and stored at −20°C for further analysis.

### Serum Parameter Measurements

Serum diamine oxidase (DOA) assays were performed with commercially available kits from Nanjing Jiancheng Bioengineering Institute (Nanjing, Jiangsu, China). Mouse tumor necrosis factor-α (TNF-α), interleukin-1β (IL-1β), and IL-6 enzyme-linked immunosorbent assay (ELISA) kits were obtained from Beijing Sizhengbai Biotechnology Co., Ltd (Beijing, China). In addition, the 3100-type automatic biochemical analyzer (Hitachi Co., Tokyo, Japan) was used to determine the concentrations of Immunoglobulin G (IgG), Urea, Creatinine (Cre), C-reactive protein (CRP), and Alanine transaminase (ALT) in serum samples.

### Histopathological Assays

Samples taken from the duodenum, jejunum, and ileum were used for histological analysis. The samples were fixed overnight in 4% paraformaldehyde and then dehydrated with different concentrations of ethanol. After dehydration, samples were embedded in paraffin and were subsequently cut into 4-μm thick sections. The prepared tissue sections were stained with hematoxylin and eosin (H&E) and sealed with a neutral gum. Villus height and crypt depth were determined by using an image processing and analysis system (Image-Pro Plus 6.0, Media Cybernetics, Inc., Bethesda, MD, USA), and a previously described calculation method were adopted (21).

### Immunofluorescence Analysis

The jejunal tissue section was deparaffinized and rinsed with distilled water for 5 min. Tissue sections were then subjected to antigen retrieval by ethylenediaminetetraacetic acid (EDTA, 1 mol/L, pH 9.0, Gooddbio Technology Co., Ltd., Wuhan, China). Before overnight incubating at 4°C with rabbit anti-ZO-1 polyclonal antibody (Gooddbio Technology Co., Ltd., Wuhan, China), sections were blocked with 3% bovine serum albumin. The sections were washed three times with PBS (pH 7.4) for 5 min each time, and then goat anti-rabbit IgG-FITC secondary antibody (Gooddbio Technology Co., Ltd., Wuhan, China) was added thereto, followed by incubation at room temperature for 50 min in the dark. Then, sections were washed three times with PBS (PH = 7.4), and the nuclei were stained with 4′-6-diamidino-2-phenylindole (DAPI, Gooddbio Technology Co., Ltd., Wuhan, China) for 10 min at room temperature in the dark. Finally, the fluorescence of the sections was visualized by a confocal scanning microscope (NIKON ECLIPSE TI-SR), and the images were taken using NIKON DS-U3 software.

### Detection of the Cell Apoptosis

The proportion of apoptotic cells in isolated jejunal mucosal cells was determined by flow cytometry (CytoFlex, Beckman Coulter, Inc., Brea, CA, USA) using PE Annexin V Apoptosis Detection Kit I (Becton, Dickinson and Company, BD Biosciences, San Jose, CA, USA). First, the jejunum was dissected, the jejunal mucosa was scraped, and then filtered through a grind and a mesh to form a cell suspension. After washing twice with ice-cold PBS, the cell sample was made into a single cell suspension of 1 × 10^6^ cells/mL. One hundred microlitre of the single cell suspension was centrifuged at 1,300 × g for 15 min to remove the supernatant, then the cells were stained with 5 μL of Annexin-V-FITC fluorescent dye at 4°C in the dark. After 10 min, add 5 μL of PI staining for 5 min at 4°C in the dark. Finally, detection of apoptotic cells was completed within 1 h after the addition of 400 μL Annexin V binding buffer (1x).

### RNA Extraction and Real-Time PCR

Total RNA was extracted from duodenal, jejunal, and ileal samples using TRIzol Reagent (TaKaRa, Dalian, China). The concentration and purity of total RNA were assayed by spectrophotometer (Beckman Coulter, DU800) at 260 and 280 nm. The ratio of absorption (260/280 nm) of samples was between 1.8 and 2.0. Then, each RNA sample was reverse-transcribed into cDNA using reverse transcriptase (Takara, Tokyo, Japan) after detection of RNA concentration and purity by spectrophotometer (Beckman Coulter, DU800). The PCR primer sequences were designed using Primer Premier 5.0 and are listed in Supplementary Table 1. Briefly, quantitative PCR was performed by QuanStudio 6 Flex Real-Time PCR detection system (Applied Biosystems, Foster City, CA, USA), with a total of 10 μL of assay solution containing 5 μL SYBR Green mix (TaKaRa, Dalian, China), 0.2 μL Rox, 3 μL deionized H_2_O, 1 μL cDNA template, and 0.4 μL each of forward and reverse primers. The comparative Ct value method was used to quantify mRNA expression relative to β-actin expression (22).

### Determination of Cysteinyl Aspartate-Specific Protease Activity

The activity of caspase-3 and caspase-9 were determined using the Cysteinyl aspartate-specific protease activity kit (Beyotime, Shanghai, China). To evaluate the caspase-3 and caspase-9 activity of the small intestine, tissue lysates were prepared after their respective treatment with various designated treatments. Assays were performed on 96-well microtiter plates by incubating 50 μL protein of tissue lysate per sample in 50 μL reaction buffer (1% NP-40, 20 mM Tris-HCl (pH 7.5), 137 mM Nad and 10% glycerol) containing 10 μL caspase-3 substrate (Ac-DEVD-*p*NA) (2 mM) or 10 μL caspase-9 substrate (Ac-LEHD-*p*NA) (2 mM). Lysates were incubated at 37 °C for 2 h. Samples were measured with the UV-1100 spectrophotometer (Shanghai, China) at an absorbance of 405 nm and 1 μg Cysteinyl aspartate-specific protease hydrolyzes Ac-DEVD-*p*NA or Ac-LEHD-*p*NA within 1 h to produce 1 nmoL of *p*NA represents U/μg.

### Statistical Analysis

The individual mouse was used as the experimental unit, and all data were expressed as mean ± standard error (SEM). Statistical analysis was carried out using two-way ANOVA followed by Bonferroni's multiple comparisons test using GraphPad Prism software (Version 7. GraphPad Software Inc., CA, USA).

## Results

### *In vitro* Assays for the Antibacterial Activity of pBD129

**Figure 1:**
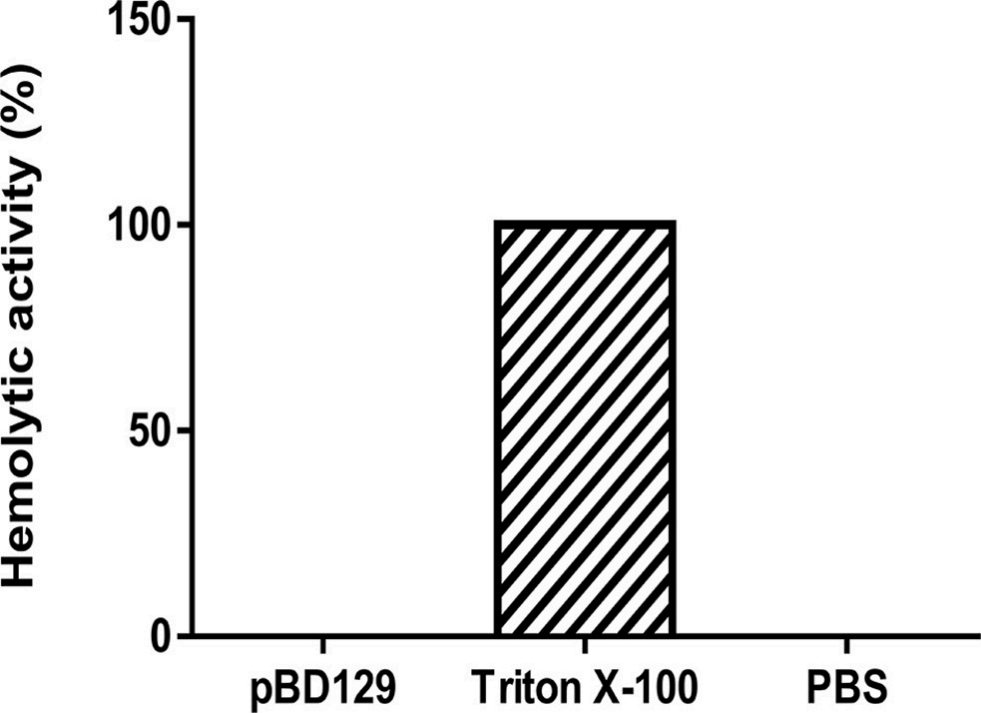
The hemolytic activity of porcine β-defensin 129. Triton X-100 and phosphate buffer saline (PBS) were selected as positive and negative controls, respectively, *n* = 3.

pBD129 (Porcine β-defensin 129) was expressed in *E. coli* BL21 (DE3) and purified by using the Ni-NTA agarose column (Supplementary Figure 1). The purity of recombinant pBD129 was analyzed with Image Lab (Bio-Rad), and the results showed that the purity of recombinant pBD129 was 90%. The purified protein was identified by mass spectrometry (LC-MS/MS). After searching the amino acid sequence of pBD129 in the NCBI database (Accession No. NP_001123447.1), we found that the sequence coverage of the two protein sequences was more than 82%, indicating that the purified protein was porcine β-defensin 129 (Supplementary Figure 2). The MIC assays were carried out to evaluate the antimicrobial activity of pBD129. As shown in Table 1, pBD129 showed significant antimicrobial activities against the *E. coli* and *Streptococcus* with a minimal inhibitory concentration (MICs) of 32 μg/mL. Moreover, we measured the hemolytic activity of the pBD129 by using whole pig blood, and found that the recombinant pBD129 had no detrimental effect on the erythrocytes at all concentrations (0–256 μg/mL) (Figure 1).

**Table 1:** Minimal inhibition concentration (MIC) of porcine β-defensin 129^a^.

**Strain**	**pBD-129 (ug/mL)**
Gram-negative bacteria	
*E.coli* DH5α	32
pathogenic *E.coli* K88+	>512
*Salmonella typhimurium* CICC14028	>512
Gram-positive bacteria	
*Streptococcus dysgalactiae* ATCC 12394	32
*Staphylococcus aureus* CICC23656	>512
*Bacillus subtilis*	>512
Fungi	
*Pichia pastoris* X33	>512

a *Values are the means of 3 replicates per treatment*.

### Effect of pBD129 on Serum Biochemical Parameters in Mice Upon LPS Challenge

The serum parameters are presented in Table 2. LPS challenge significantly increased the serum concentrations of inflammatory cytokines such as the IL-1β, IL-6, and TNF-α (*P* < 0.05). However, pBD129 injection significantly decreased the serum concentrations of these inflammatory cytokines upon LPS challenge (*P* < 0.05). No significant changes of serum inflammatory cytokines were observed in mice without being LPS challenged (*P* < 0.05). Additionally, LPS challenge significantly increased the serum concentrations of ALT, CRP, Cre, and urea (*P* < 0.05). Amongst the LPS-challenged groups, pBD129 injection at a high dose (8 mg/kg) significantly decreased the serum concentrations of ALT, CRP, Cre, and urea (*P* < 0.05). Moreover, pBD129 injection at a lower lose (4 mg/kg) can also decrease the serum concentrations of urea and Cre (*P* < 0.05). Interestingly, mice with LPS challenge showed an acute reduction in serum IgG concentration (*P* < 0.01), but pBD129 injection at 8 mg/kg significantly increased the serum IgG concentration (*P* < 0.01).

**Table 2:** Porcine ionsBACTERIAf pameliorated the Biochemical Parameters of Serum during Bacterial Endotoxin-induced pathology^1^.

**Item^2^**	**Treatment^3^**						***P*-value^4^**		
	**Control**	**L-129**	**H-129**	**LPS**	**L-129 + LPS**	**H-129 + LPS**	**B**	**V**	B*V
IL-1β (pg/mL)	29.98 ± 9.76^b^	37.13 ± 10.12^b^	32.02 ± 12.48^b^	198.86 ± 18.52^a^	35.40 ± 6.15^b^	38.88 ± 6.54^b^	<0.0001	<0.0001	<0.0001
IL-6 (pg/mL)	23.25 ± 0.72^c^	24.71 ± 1.14^c^	24.02 ± 0.83^c^	1934.21 ± 13.16^a^	199.11 ± 39.29^b^	85.01 ± 12.79^c^	<0.0001	<0.0001	<0.0001
TNF-α (pg/mL)	28.89 ± 0.74^b^	32.84 ± 1.11^b^	31.62 ± 2.31^b^	73.32 ± 5.56^a^	40.79 ± 1.67^b^	35.68 ± 1.40^b^	<0.0001	<0.0001	<0.0001
ALT (mmol/L)	43.25 ± 2.21^b^	43.50 ± 6.08^b^	37.50 ± 1.19^b^	83.50 ± 5.06^a^	80.00 ± 6.87^a^	36.75 ± 2.75^b^	<0.0001	<0.0001	0.0004
CRP (mg/L)	0.66 ± 0.22^b^	0.75 ± 0.09^b^	0.41 ± 0.16^b^	3.83 ± 0.66^a^	3.25 ± 0.45^a^	1.38 ± 0.15^b^	0.0029	<0.0001	0.0175
Cre (mmol/L)	7.33 ± 0.08^c^	7.70 ± 0.16^bc^	8.86 ± 0.09^bc^	11.00 ± 0.72^a^	8.99 ± 0.34^b^	9.08 ± 0.19^b^	0.0675	<0.0001	0.0003
urea (mmol/L)	7.99 ± 0.13^d^	7.95 ± 0.25^d^	8.10 ± 0.26^d^	20.05 ± 0.18^a^	10.81 ± 1.14^c^	13.28 ± 0.22^b^	<0.0001	<0.0001	<0.0001
IgG (g/L)	0.58 ± 0.05^b^	0.66 ± 0.08^a^^b^	0.88 ± 0.06^a^	0.18 ± 0.05^c^	0.44 ± 0.07^bc^	0.55 ± 0.03^b^	<0.0001	<0.0001	0.2915
DAO (U/L)	24.15 ± 0.86^b^	25.07 ± 0.88^b^	27.82 ± 1.61^a^^b^	31.44 ± 0.60^a^	27.46 ± 1.52^a^^b^	25.46 ± 0.18^b^	0.3584	0.0159	0.0025

*Different lowercase letters indicate statistically significant differences from each other (P < 0.05)*.

1 *Values of the IgG, Cre, CRP, ALT, and UREA are 4 replicates per treatment; Values of the IL-1β, IL-6, and TNF-α are 5 replicates per treatment. Values of the DAO is 3 replicates per treatment*.

2 *IgG, Immunoglobulin G; Cre, creatinine; CRP, C-reactive protein; ALT, Alanine transaminase; DAO, diamine oxidase; IL-1β, interleukin-1β; IL-6, interleukin-6; TNF-α, tumor necrosis factor-α*.

3 *Control, 200 μL sterile saline; LPS, 200 μL Lipopolysaccharide; L-pBD129, 200 μL of 0.6 mg/ml porcine β-defensin; H-pBD129, 200 μL of 1.2 mg/mL porcine β-defensin 129; L-pBD129 + LPS, 200 μL of 0.6 mg/mL pBD129 pretreated followed by LPS treated; H-pBD129 + LPS, 200 μL of 1.2 mg/mL pBD129 pretreated followed by LPS treated*.

4 B is the main effect of porcine β-defensin 129; V is the main effect of LPS infection; B*V is the interaction effect of the two main factors.

### Effect of pBD129 on Intestinal Morphology, Permeability, and Distribution of the Major Tight Junction-Associated Protein ZO-1

**Figure 2:**
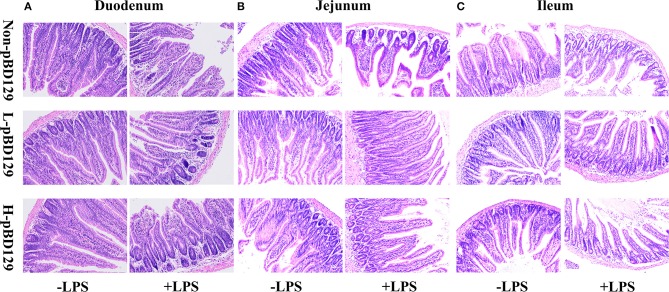
Histological evaluation of small intestine tissue after exposure to pBD129 (H&E; × 200). **(A)** Representative H&E stained sections from the duodenum. **(B)** Representative H&E stained sections from the jejunum. **(C)** Representative H&E stained sections from the ileum. Non-pBD129, 200 μL Sterilized saline; L-pBD129, 200 μL of 0.6 mg/ml porcine β-defensin 129; H-pBD129, 200 μL of 1.2 mg/ml porcine β-defensin 129, *n* = 3/group.

**Figure 3:**
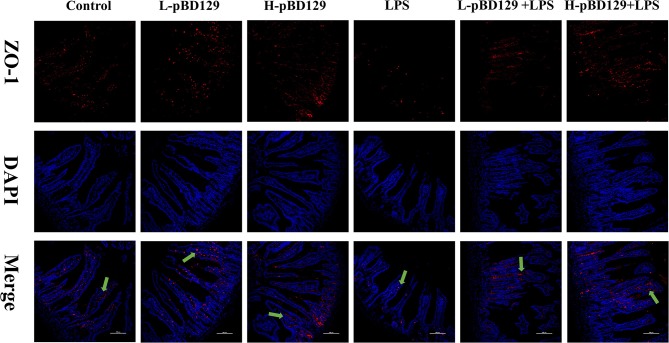
Evaluation of the localization of ZO-1 and DAPI (DNA) in the jejunum of mice by immunofluorescence. ZO-1 protein (red), DAPI staining (blue), and pooled ZO-1 protein and DAPI are provided. The scale bar represents 100 μm. The green arrows mark jejunum positive for ZO-1 expression. Control, 200 μL sterile saline; LPS, 200 μL Lipopolysaccharide; L-pBD129, 200 μL of 0.6 mg/ml porcine β-defensin; H-pBD129, 200 μL of 1.2 mg/mL porcine β-defensin 129; L-pBD129 + LPS, 200 μL of 0.6 mg/mL pBD129 pretreated followed by LPS treated; H-pBD129 + LPS, 200 μL of 1.2 mg/mL pBD129 pretreated followed by LPS treated.

LPS challenge resulted in atrophy of the intestinal mucosa (Figure 2). As compared to the control group (challenged by sterile saline), the LPS-challenged mice have a shedding epithelium and shortened villi in the small intestine (Table 3). However, the villus height in the jejunum and ileum were significantly elevated by pBD129 in the LPS-challenged mice (*p* < 0.05). Moreover, pBD129 significantly decreased the crypt depth and elevated the ratio of villus height/crypt depth in the small intestine (*p* < 0.05). To investigate the intestinal permeability, the serum DAO concentrations were determined (Table 2). We show that LPS challenge acutely increased the serum DAO concentrations (*p* < 0.01). However, pBD129 treatment at a higher dose (8 mg/kg) significantly decreased the serum DAO concentration in LPS-challenge mice (*P* < 0.05). Importantly, we explored the distribution of the major tight junction-associated protein ZO-1 in jejunum by immunofluorescence analysis, and found that the localization of ZO-1 protein in the jejunum was significantly changed after LPS challenge (Figure 3). As compared to the control group, LPS challenge has resulted in decreased abundance of ZO-1 protein in the tight junction region, indicating the disruption of the tight junction. In contrast, the abundance of ZO-1 protein was significantly elevated and localized to the apical intercellular region of the intestinal epithelium in mice treated by pBD129.

**Table 3:** Effects of Porcine β-defensin 129 on the intestinal morphology of mice^1^.

**Item^2^**	**Treatment^3^**						***P*-value^4^**		
	**Control**	**L-129**	**H-129**	**LPS**	**L-129+LPS**	**H-129+LPS**	**B**	**V**	B*V
Duodenum									
VH, μm	478.10 ± 2.54^a^	466.27 ± 1.68^a^	471.79 ± 6.25^a^	413.88 ± 17.25^b^	445.34 ± 7.10^ab^	428.28 ± 16.51^ab^	0.6529	<0.0001	0.1510
CD, μm	136.88 ± 5.20^b^	140.10 ± 2.77^b^	138.85 ± 0.81^b^	181.04 ± 3.30^a^	153.03 ± 3.30^b^	146.39 ± 4.02^b^	0.0005	<0.0001	0.0001
VH/CD	3.51 ± 0.12^a^	3.33 ± 0.08^a^	3.40 ± 0.06^a^	2.29 ± 0.09^c^	2.92 ± 0.09^b^	2.92 ± 0.05^b^	0.0113	<0.0001	0.0002
Jejunum									
VH, μm	398.03 ± 9.50^a^	370.33 ± 11.47^ab^	392.49 ± 11.46^a^	253.60 ± 13.07^c^	342.34 ± 13.07^ab^	325.02 ± 14.04^b^	0.0254	<0.0001	0.0005
CD, μm	117.51 ± 2.08^b^	114.73 ± 2.84^b^	114.86 ± 1.69^b^	142.62 ± 6.95^a^	125.35 ± 3.42^ab^	118.64 ± 2.83^b^	0.0059	0.0004	0.0302
VH/CD	3.39 ± 0.07^a^	3.23 ± 0.09^a^	3.42 ± 0.12^a^	1.79 ± 0.14^c^	2.73 ± 0.08^b^	2.74 ± 0.10^b^	0.0003	<0.0001	<0.0001
Ileum									
VH, μm	224.83 ± 3.22^ab^	221.24 ± 3.70^ab^	228.64 ± 2.99^a^	168.30 ± 6.82^c^	208.01 ± 3.76^ab^	207.70 ± 1.55^b^	<0.0001	<0.0001	<0.0001
CD, μm	86.04 ± 2.72^c^	93.24 ± 1.86^c^	93.82 ± 1.17^c^	120.91 ± 2.02^a^	105.09 ± 0.54^b^	103.85 ± 1.00^b^	0.0247	<0.0001	<0.0001
VH/CD	2.62 ± 0.12^a^	2.38 ± 0.07^a^	2.44 ± 0.05^a^	1.39 ± 0.06^c^	1.98 ± 0.05^b^	2.0 ± 0.03^b^	0.0150	<0.0001	<0.0001

*Different lowercase letters indicate statistically significant differences from each other (P < 0.05)*.

1 *Values are the means of 3 replicates per treatment*.

2 *VH villus height, CD crypt depth, VH/CD the ratio of villus height and crypt depth*.

3 *Control, 200 μL sterile saline; LPS, 200 μL Lipopolysaccharide; L-pBD129, 200 μL of 0.6 mg/ml porcine β-defensin; H-pBD129, 200 μL of 1.2 mg/mL porcine β-defensin 129; L-pBD129 + LPS, 200 μL of 0.6 mg/mL pBD129 pretreated followed by LPS treated; H-pBD129 + LPS, 200 μL of 1.2 mg/mL pBD129 pretreated followed by LPS treated*.

4 B is the main effect of Porcine β-defensin 129; V is the main effect of LPS infection; B*V is the interaction effect of the two main factors.

### Effect of pBD129 on Intestinal Epithelium Cell Apoptosis

**Figure 4:**
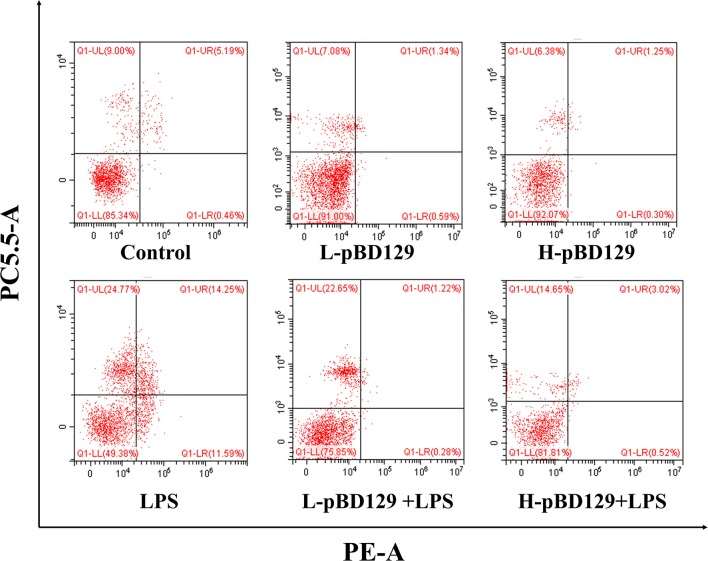
Percentage of apoptotic cells in the jejunal mucosal of mouse. Frames were divided into four quadrants: Q1–UL represents necrotic cells. Q1–UR represents late-stage apoptotic cells. Q1–LL represents normal cells. Q1–LR represents early-stage apoptotic cells. Control, 200 μL sterile saline; LPS, 200 μL Lipopolysaccharide; L-pBD129, 200 μL of 0.6 mg/ml porcine β-defensin; H-pBD129, 200 μL of 1.2 mg/mL porcine β-defensin 129; L-pBD129 + LPS, 200 μL of 0.6 mg/mL pBD129 pretreated followed by LPS treated; H-pBD129 + LPS, 200 μL of 1.2 mg/mL pBD129 pretreated followed by LPS treated. *n* = 3/group.

We found that necrotic apoptosis in the intestinal mucosa was significantly changed after LPS challenge (Figure 4). As compared to the control group, LPS challenge has resulted in elevated necrotic apoptosis in the intestinal mucosa. In contrast, the necrotic apoptosis was significantly decreased in the intestinal epithelium in mice treated by pBD129. In addition, as shown in Figure 4 and Table 4, LPS challenge significantly increased the percentage of the early-stage apoptotic cells and the total apoptotic cells in the intestinal mucosa (*P* < 0.05). However, pBD129 significantly reduced the percentage of the early-stage apoptotic cells and the total apoptotic cells in the LPS-challenged mice (*P* < 0.05). Interestingly, the caspase-3 and caspase-9 activities in the small intestine were measured and, as shown in Table 5, LPS challenge significantly increased the activity of caspase 3 and 9. However, pBD129 reduced their activities in the LPS-challenged mice (*P* < 0.05).

**Table 4:** Effects of Porcine β-defensin 129 on the Jejunal mucosal apoptosis of mice^1^.

**Item^2^**	**Treatment^3^**						***P*-value^4^**		
	**Control**	**L-129**	**H-129**	**LPS**	**L-129+LPS**	**H-129+LPS**	**B**	**V**	B*V
EP	0.92 ± 0.26^b^	1.60 ± 0.63^b^	0.21 ± 0.05^b^	7.81 ± 2.22^a^	1.28 ± 0.91^b^	0.51 ± 0.12^b^	0.0056	0.0177	0.0071
LP	5.70 ± 0.26	2.07 ± 1.33	1.36 ± 0.06	7.81 ± 2.22	2.91 ± 1.33	4.63 ± 1.32	0.0132	0.0768	0.6580
TP	6.62 ± 0.51^b^	3.67 ± 1.26^b^	1.58 ± 0.03^b^	21.23 ± 2.49^a^	4.18 ± 1.37^b^	5.15 ± 1.22^b^	<0.0001	0.0001	0.0006

*Different lowercase letters indicate statistically significant differences from each other (P < 0.05)*.

1 *Values are the means of 3 replicates per treatment*.

2 *EP, Early-stage apoptotic cell percentage; LP, Late-stage apoptotic cell percentage; TP, Total apoptotic cell percentage*.

3 *Control, 200 μL sterile saline; LPS, 200 μL Lipopolysaccharide; L-pBD129, 200 μL of 0.6 mg/ml porcine β-defensin; H-pBD129, 200 μL of 1.2 mg/mL porcine β-defensin 129; L-pBD129 + LPS, 200 μL of 0.6 mg/mL pBD129 pretreated followed by LPS treated; H-pBD129 + LPS, 200 μL of 1.2 mg/mL pBD129 pretreated followed by LPS treated*.

4 B is the main effect of Porcine β-defensin 129; V is the main effect of LPS infection; B*V is the interaction effect of the two main factors.

**Table 5:** Effects of Porcine β-defensin 129 on the intestinal cysteinyl aspartate-specific protease activity of mice^1^.

**Item^2^**	**Treatment^3^**						***P*-value^4^**		
	**Control**	**L-129**	**H-129**	**LPS**	**L-129+LPS**	**H-129+LPS**	**B**	**V**	B*V
Duodenum									
Cas-3, U/μg	178.84 ± 9.49^c^	177.81 ± 9.98^c^	187.37 ± 0.74^c^	555.12 ± 26.42^a^	463.69 ± 33.86^ab^	377.76 ± 4.54^b^	<0.001	0.002	0.001
Cas-9, U/μg	292.39 ± 26.81^c^	282.09 ± 58.13^c^	273.20 ± 43.58^c^	831.71 ± 22.22^a^	761.70 ± 55.09^ab^	569.64 ± 50.40^b^	<0.001	0.02	0.05
Jejunum									
Cas-3, U/μg	701.59 ± 30.64^b^	688.20 ± 17.36^b^	813.20 ± 83.49^ab^	1268.81 ± 229.91^a^	779.77 ± 82.94^ab^	736.09 ± 33.63^ab^	0.05	0.08	0.03
Cas-9, U/μg	466.25 ± 10.51^b^	494.21 ± 16.43^b^	516.11 ± 15.09^b^	708.94 ± 41.81^a^	530.32 ± 53.65^b^	503.62 ± 13.17^b^	0.004	0.04	0.003
Ileum									
Cas-3, U/μg	312.39 ± 62.77^bc^	193.70 ± 19.08^c^	189.52 ± 7.00^c^	630.45 ± 53.94^a^	459.95 ± 40.12^ab^	291.49 ± 38.29^bc^	<0.001	<0.001	0.06
Cas-9. U/μg	652.58 ± 77.00^bc^	427.55 ± 34.24^c^	479.06 ± 53.22^c^	1038.46 ± 92.64^a^	848.28 ± 31.35^ab^	694.09 ± 27.73^bc^	<0.001	0.002	0.21

*Different lowercase letters indicate statistically significant differences from each other (P < 0.05)*.

1 *Values of the Cas-3 and Cas-9 are 3 replicates per treatment*.

2 *Cas-3, Cysteinyl aspartate-specific protease-3; Cas-9, Cysteinyl aspartate-specific protease-9*.

3 *Control, 200 μL sterile saline; LPS, 200 μL Lipopolysaccharide; L-pBD129, 200 μL of 0.6 mg/ml porcine β-defensin; H-pBD129, 200 μL of 1.2 mg/mL porcine β-defensin 129; L-pBD129 + LPS, 200 μL of 0.6 mg/mL pBD129 pretreated followed by LPS treated; H-pBD129 + LPS, 200 μL of 1.2 mg/mL pBD129 pretreated followed by LPS treated*.

4 B is the main effect of porcine β-defensin 129; V is the main effect of LPS infection; B*V is the interaction effect of the two main factors.

### Effect of pBD129 on Critical Genes Related to Inflammatory Response, Intestinal Barrier Functions, and Cell Apoptosis

**Figure 5:**
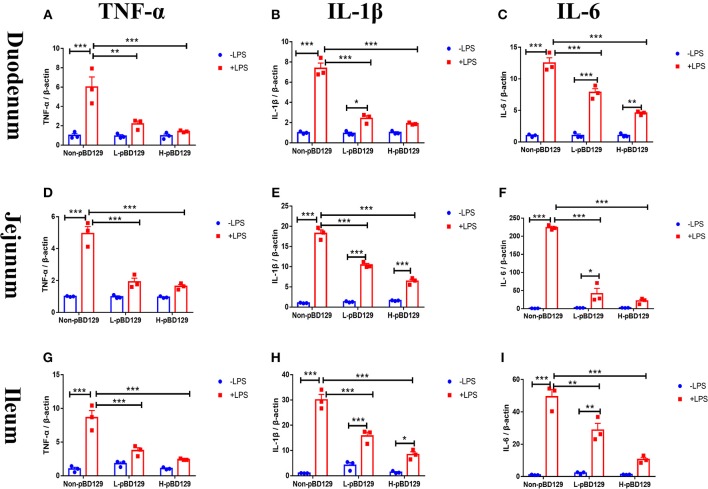
Real time PCR analysis of TNF-α **(A)**, IL-1β **(B)**, IL-6 **(C)** mRNA abundance in Duodenum; Real time PCR analysis of TNF-α **(D)**, IL-1β **(E)**, IL-6 **(F)** mRNA abundance in Jejunum; Real time PCR analysis of TNF-α **(G)**, IL-1β **(H)**, IL-6 **(I)** mRNA abundance in Ileum. IL-1β, interleukin-1β; IL-6, interleukin-6; TNF-α, tumor necrosis factor-α. Non-pBD129, 200 μL Sterilized saline; L-pBD129, 200 μL of 0.6 mg/ml porcine β-defensin 129; H-pBD129, 200 μL of 1.2 mg/ml porcine β-defensin 129. A-I, *n* = 3/group. *P < 0.05, **P < 0.01, ***P < 0.001. Results are given as means ± SEM. Two-way ANOVA followed by Bonferroni's multiple comparisons test.

**Figure 6:**
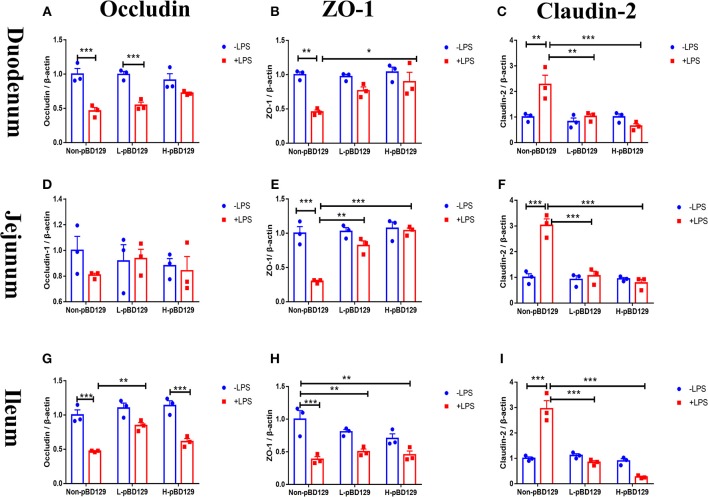
Determination of relative changes in gene expression of TJ proteins in duodenum by real-time PCR analysis. **(A)** Occludin, **(B)** ZO-1, **(C)** Claudin-2. Determination of relative changes in gene expression of TJ proteins in Jejunum by real-time PCR analysis. **(D)** Occludin, **(E)** ZO-1, **(F)** Claudin-2; Determination of relative changes in gene expression of TJ proteins in Ileum by real-time PCR analysis. **(G)** Occludin, **(H)** ZO-1, **(I)** Claudin-2. ZO-1, Zonula occludens-1. A-I, *n* = 3/group. *P < 0.05, **P < 0.01, ***P < 0.001. Results are given as means ± SEM. Two-way ANOVA followed by Bonferroni's multiple comparisons test.

**Figure 7:**
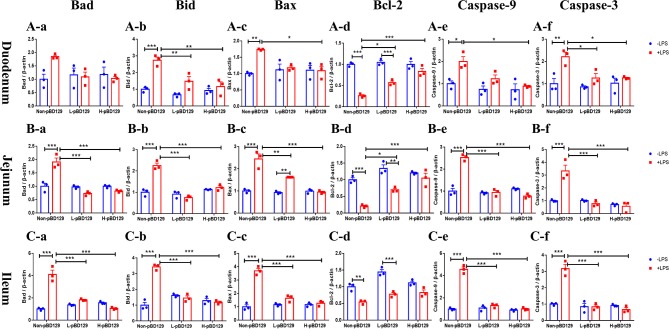
Real time PCR analysis of Bad **(A-a)**, Bid **(A-b)**, Bax **(A-c)**, Bcl-2 **(A-d)**, Caspase-9 **(A-e)**, Caspase-3 **(A-f)** mRNA abundance in duodenum; Real time PCR analysis of Bad **(B-a)**, Bid **(B-b)**, Bax **(B-c)**, Bcl-2 **(B-d)**, Caspase-9 **(B-e)**, Caspase-3 **(B-f)** mRNA abundance in jejunum; Real time PCR analysis of Bad **(C-a)**, Bid **(C-b)**, Bax **(C-c)**, Bcl-2 **(C-d)**, Caspase-9 **(C-e)**, Caspase-3 **(C-f)** mRNA abundance in Ileum. Bad, Bcl-2 antagonist of cell death; BAX, B-cell lymphoma-2-associated X protein; Bid, BH3-interacting domain death agonist; BCL2, B-cell lymphoma-2; caspase-3, cysteinyl aspartate-specific proteinase-3; caspase-9, cysteinyl aspartate-specific proteinase-9. **(A-a)**–**(C-f)**, *n* = 3/group. *P < 0.05, **P < 0.01, ***P < 0.001. Results are given as means ± SEM. Two-way ANOVA followed by Bonferroni's multiple comparisons test.

As shown in Figure 5, LPS challenge significantly elevated the expression levels of inflammatory cytokines such as the IL-6, IL-1β, and TNF-α in the small intestine (*P* < 0.01). However, pBD129 significantly decreased their expression levels in the LPS-challenged mice (*P* < 0.01). The expression levels of critical tight junction proteins such as the ZO-1, Occludin, and Claudin-2 were determined. As shown in Figure 6, LPS challenge has resulted in down-regulation of ZO-1 and Occludin in the small intestine (*P* < 0.01). However, pBD129 significantly elevated their expression levels in the duodenum and jejunum mucosa. In contrast, pBD129 decreased the expression level of Claudin-2 in the small intestine (*P* < 0.01). We also investigated the expression levels of critical apoptotic-related genes. As shown in Figure 7, LPS challenge down-regulated the expression of Bcl-2, but significantly elevated the expression levels of apoptotic genes such as the Bad, Bid, and Bax in the small intestinal mucosa (*P* < 0.05). However, pBD129 not only elevated the expression of Bcl-2, but also down-regulated the expression levels of the three critical apoptotic genes (*P* < 0.05). Moreover, LPS challenge resulted in up-regulation of caspase-3 and caspase-9 in the small intestine (*P* < 0.05). However, pBD129 significantly decreased their expression levels in the LPS-challenged mice (*P* < 0.05).

## Discussion

Apoptosis of intestinal epithelial cells induced by pathogens disrupts intestinal barrier functions (23). In recent years, the β-defensins has attracted considerable research interest since it has been reported to play a critical role in the modulating the adaptive immunity and improving the intestinal barrier functions (24, 25). The pBD129 is a newly discovered porcine beta-defensin, which is highly expressed in the epithelial cells of the gastrointestinal mucosa (26, 27). In this study, we explored the role of pBD129 in regulating the inflammatory responses and intestinal epithelium barrier functions in mice.

The pBD129 was successfully expressed in *E. coli* BL21 (DE3) and the soluble proteins in the periplasmic space were purified. A significant degree of overlap (82%) was observed between the proteins identified in the LC-MS/MS data sets, indicating that the purified protein was porcine β-defensin 129. Antimicrobial activity assays showed that pBD129 has significant antimicrobial activity against the gram-positive bacteria (*Streptococcus*) and gram-negative bacteria (*E. coli* DH5α). The result is also consistent with previous studies on the porcine β-defensins (28, 29). Both indicated that the porcine β-defensins has a broad antibacterial spectrum. Moreover, we found that the pBD129 has a weak hemolytic activity, indicating that it is harmless to humans and animals, and may be tentatively used as a substitute for conventionally used antibiotics.

Lipopolysaccharide (LPS) is an important structural component of the outer membrane of gram-negative bacteria which triggers the systemic inflammation and induces damage of target organs such as kidneys, liver, and intestinal mucosa (30). In the present study, the serum concentrations of inflammatory cytokines such as the IL-1β, IL-6, and TNF-α were both elevated upon LPS challenge, indicating the success of model construction. Interestingly, pBD129 treatment significantly decreased the serum concentrations of these inflammatory cytokines which suggested the β-defensins may act as a negative regulator for inflammatory responses. This result is consistent with previous studies on a variety of animal species (31, 32). Moreover, pBD129 treatment at a high dose (8 mg/kg) significantly decreased the serum concentrations of ALT, CRP, Cre, and urea, which has been widely used as biological markers of kidney and hepatic functionality (33). Additionally, pBD129 treatment (8 mg/kg) significantly increased the serum IgG concentration, which is consistent with previous findings that the β-defensins can act as a positive immune regulator for animals (34).

The intestinal epithelium provides a protective barrier, preventing both pathogenic, and commensal bacteria from escaping from the intestinal lumen. But some enteric pathogens can induce permeability defects in gut epithelia by altering tight junction proteins, which allows the translocation of toxins via the mucosa to access the whole body, subsequently destroying the intestinal mucosal homeostasis (35, 36). Disruption of the intestinal epithelium impairs the nutrient digestion and absorption (37). In the present study, LPS challenge significantly decreased the villus height in the small intestine. However, pBD129 significantly elevated the villus height in the LPS-challenged mice. This is probably due to the reduced inflammatory cytokines, since the IL-1β, IL-6, and TNF-α were found to induce atrophy of intestinal mucosa and disruption of intestinal functions (38–40). The DAO is a catalytic enzyme which is mainly synthesized in the digestive tract and involved in the metabolism, oxidation, and inactivation of histamine and other polyamines such as putrescine and spermidine in animals (41). Importantly, the serum DAO concentration has been widely used as a biomarker of the intestinal permeability since it can be released into the blood circulation (42). In the present study, LPS challenge significantly elevated the serum DAO concentration, indicating the disruption of the intestinal epithelium barriers. However, pBD129 treatment at 8 mg/kg significantly decreased the serum DAO concentration in LPS-challenge mice, indicating a protective effect of the β-defensins on intestinal mucosal integrity.

The intestinal epithelial cells (IECs) are connected in the lateral membrane by forming the tight junction (TJs) (43). TJs are mainly composed of cytoplasmic scaffold proteins such as ZO-1, transmembrane proteins including claudins, and attachment adhesion molecules (JAM) (5), which controls the paracellular permeability of small molecules (44). Previous studies have indicated that inflammatory stress (i. LPS challenge) significantly decreased the abundance of TJ proteins (45, 46). A similar result was observed in the present study. However, we found that the abundance of ZO-1 protein was significantly elevated and localized to the apical intercellular region of the intestinal epithelium in mice after pBD129 treatment. The result is consistent with a previous study on porcine beta-defensin-2 (PBD-2) in DSS-treated mouse model.

Apoptosis is a form of physiological cell death that is important for the renewal of intestinal mucosa cells. In severe intestinal pathology, breakdown of intestinal mucosa via accelerated apoptosis increases intestinal permeability (47, 48). Previous studies have indicated that infections or stresses can increase intestinal epithelial cell apoptosis (49, 50). In the present study, LPS challenge increased the percentage of the apoptotic cells in the intestinal mucosa. However, pBD129 treatment significantly reduced the percentage of the early-stage apoptotic cells and the total apoptotic cells in the intestinal mucosa from LPS-challenged mice. This is also probably due to the decreased inflammatory cytokines after pBD129 treatment, since the IL-1β and TNF-α were found to induce apoptosis via intrinsic mitochondrial apoptotic pathway (51–53).

To gain insights into the mechanisms behind the pBD129 modulated intestinal barrier functions, we explored the expression levels of some critical molecules involved in the regulation of inflammatory response and apoptosis. Interestingly, the pBD129 was found to significantly decrease the expression levels of several critical inflammatory cytokines (i.e., IL-1β, IL-6, and TNF-α) and tight junction proteins (i.e., ZO-1 and Occludin) in the intestinal mucosa. The result is consistent with previous studies using different animal species (54, 55). The Bcl-2 is localized to the outer membrane of mitochondria, where it plays a critical role in promoting cellular survival and inhibiting the actions of pro-apoptotic proteins (56). In the present study, pBD129 treatment significantly elevated the expression levels of Bcl-2 and down-regulated the expression levels of critical apoptotic genes (Bad, Bid, Bax, caspase-3, and caspase-9) in the intestinal mucosa of LPS-challenged mice. The Bad, Bid, and Bax contributed to programmed cell death by inducing mitochondrial cytochrome *c* release, which activates caspase-9 and then caspase-3 (57, 58). The caspase-3 and caspase-9 are responsible for executing cell death during the demolition phase of apoptosis (59, 60), and we also found that pBD129 treatment significantly reduced the caspase-3 and caspase-9 activities in the small intestine of LPS-challenged mice. For all gene expression experiments (Figures 5–7), pBD129 has no dose-dependent effect, probably because the range of dose selection is not large, and a broad range of doses could be considered in the further study. In the present study, pBD129 significantly decreased the expression levels of caspase-3 and caspase-9 in the intestinal mucosa of LPS-challenged mice. These results offer a molecular basis for the pBD129 mediated cell apoptosis in the intestinal mucosa.

In conclusion, the pBD129 attenuates bacterial endotoxin-induced inflammatory responses and intestinal mucosa atrophy by reducing the secretion of inflammatory cytokines and the apoptosis of intestinal epithelial cells. Our results suggested a novel function of the mammalian defensins, and the anti-bacterial and anti-inflammatory properties of pBD129 may allow it a potential agent to prevent or alleviate the LPS-induced inflammation and damage of the intestinal epithelium barriers.

## Data Availability Statement

All datasets generated for this study are included in the manuscript/Supplementary Files.

## Ethics Statement

This study was approved by the Animal Welfare Committee of Sichuan Agricultural University (No. 20180718).

## Author Contributions

KX and HX performed most of the experiments. GS conducted the preparation of the pBD129 protein experiment. KX was also in charge of preparing the manuscript. JH contributed to study design and revised the manuscript. DC, BY, XM, ZH, JY, JL, PZ and YL contributed to the sample collection.

### Conflict of Interest

The authors declare that the research was conducted in the absence of any commercial or financial relationships that could be construed as a potential conflict of interest.
