# Supplementary material for: β-Defensin 129 Attenuates Bacterial Endotoxin-Induced Inflammation and Intestinal Epithelial Cell Apoptosis
Source: Front Immunol. 2019 Oct 4;10:2333. doi: 10.3389/fimmu.2019.02333 (PMC6787771; doi:10.3389/fimmu.2019.02333)
Supplement: Supplementary file 1 [file Data_Sheet_1.docx]

**SUPPLEMENTARY MATERAL**

**SUPPLEMENTARY FIGURE 1** The SDS-PAGE of the recombinant PBD129 protein. Lane 1, *E. coli* Origami B (DE3)-pET32a(+) did not induce for 8 h at 28 ℃; Lane 2, *E. coli* Origami B (DE3)-pET32a(+) induced by 1 mM IPTG for 8 h at 28 ℃; Lane 3, *E. coli* Origami B (DE3)-pET32a(+)-PBD1129 did not induce for 8 h at 28 ℃; Lane 4, *E. coli* Origami B (DE3)-pET32a(+)-PBD129 induced for 8 h at 28 ℃; Lane 5, the recombinant pBD129 protein purified by Ni^2+^ chelating affinity column (37.2 kDa); Lane M, 150 KDa protein marke.

**SUPPLEMENTARY FIGURE 2** Mass spectrometry results of porcine β-defensin 129. Results were obtained by searching for uniport-Sus-scrofa (68,152 sequences; 25,615,784 resides), and found that sequence coverage is 82% (Matched peptides shown in Bold Red).

**Supplementary Table 1.** Primers for real-time PCR

| **Gene** | **Primer Sequences** | **Product Size (bp)** | **GenBank Accession No.** |
| --- | --- | --- | --- |
| *ZO-1* | F: 5’-CCCGGACTTTTGTCCCACTT-3’ | 105 | NM_009386 |
|  | R: 5’-CCACCGTCCGCATAAACATC-3’ |  |  |
| *Occludin* | F: 5'-CCGGCCGCCAAGGTTC-3’ | 103 | NM_008756.2 |
|  | R: 5’-ACTTTCAAAAGGCCTCACGGA-3’ |  |  |
| *Claudin-2* | F: 5’-ATGGCATCCAGCAGAATACA-3’ | 131 | NM_016675 |
|  | R: 5’-ACCCACAAATGTACGGGAAT-3’ |  |  |
| *Bax* | F: 5’-AGACAGGGGCCTTTTTGCTAC-3’ | 137 | NM_007527.3 |
|  | R: 5’-AATTCGCCGGAGACACTCG-3’ |  |  |
| *Bcl-2* | F: 5’-GTGGATGACTGAGTACCTGAACC-3’ | 120 | NM_009741.5 |
|  | R: 5’-AGCCAGGAGAAATCAAACAGAG-3’ |  |  |
| *Bid* | F: 5’-GCCGAGCACATCACAGACC-3’ | 226 | NM_007544.3 |
|  | R: 5’-TGGCAATGTTGTGGATGATTTCT-3’ |  |  |
| *Bad* | F: 5’-TGAGCCGAGTGAGCAGGAA-3’ | 154 | NM_007522.3 |
|  | R: 5’-GCCTCCATGATGACTGTTGGT-3’ |  |  |
| *Caspase-9* | F: 5’-TCCTGGTACATCGAGACCTTG-3’ | 109 | NM_001355176.1 |
|  | R: 5’-AAGTCCCTTTCGCAGAAACAG-3’ |  |  |
| *Caspase-3* | F: 5’-ACAGCACCTGGTTACTATTC-3’ | 225 | NM_009810.3 |
|  | R: 5’-CAGTTCTTTCGTGAGCAT-3’ |  |  |
| *IL-1ß* | F: 5’-ACCTGTGTCTTTCCCGTGG-3’ | 162 | NM_008361 |
|  | R: 5’-TCATCTCGGAGCCTGTAGTG-3’ |  |  |
| *IL-6* | F: 5’-ATCCAGTTGCCTTCTTGGGACTGA-3’ | 134 | NM_031168.2 |
|  | R: 5’-TAAGCCTCCGACTTGTGAAGTGGT-3’ |  |  |
| *TNF-α* | F: 5’-TCTCATGCACCACCATCAAGGACT-3’ | 92 | NM_013693.3 |
|  | R: 5’-ACCACTCTCCCTTTGCAGAACTCA-3’ |  |  |
| *ß-actin* | F: 5’-GCAAGCAGGAGTACGATGAGT-3’ | 86 | NM_007393.5 |
|  | F: 5’-GGTGTAAAACGCAGCTCAGTA-3’ |  |  |

The sequences in Table 1 are available on GenBank (http://www.ncbi.nlm.nih.gov/ nuccore/) under the accession numbers. F, forward; R, reverse.
