# Supplementary figures and images for: β-Defensin 129 Attenuates Bacterial Endotoxin-Induced Inflammation and Intestinal Epithelial Cell Apoptosis
Source: Front Immunol. 2019 Oct 4;10:2333. doi: 10.3389/fimmu.2019.02333 (PMC6787771; doi:10.3389/fimmu.2019.02333)

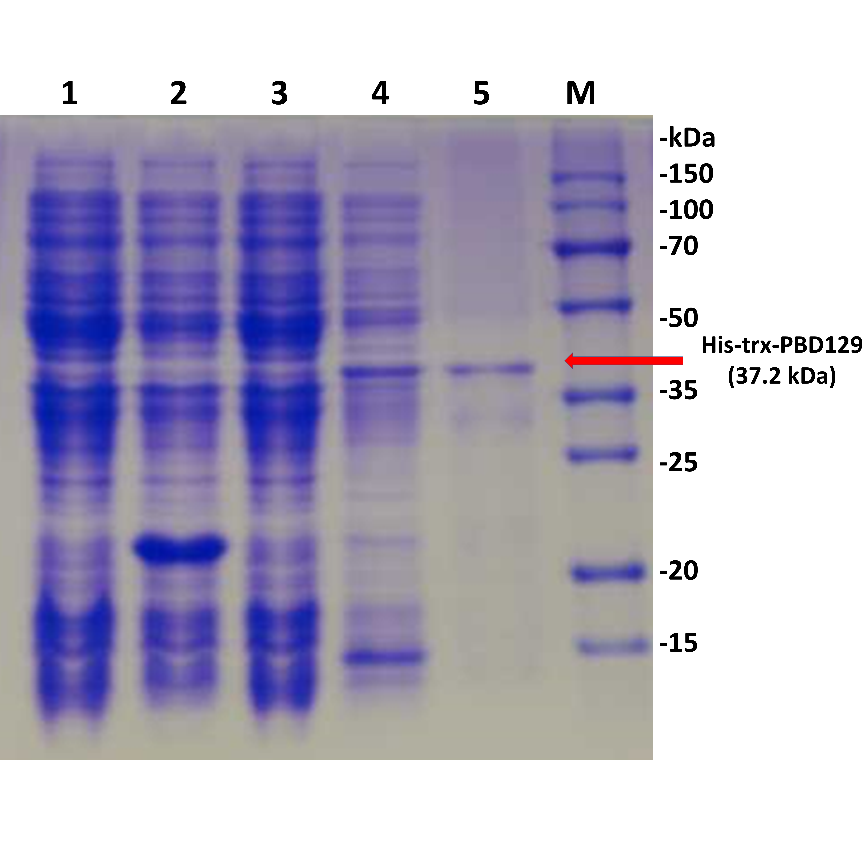

Supplement: Supplementary file 2 [file Image_1.TIF]

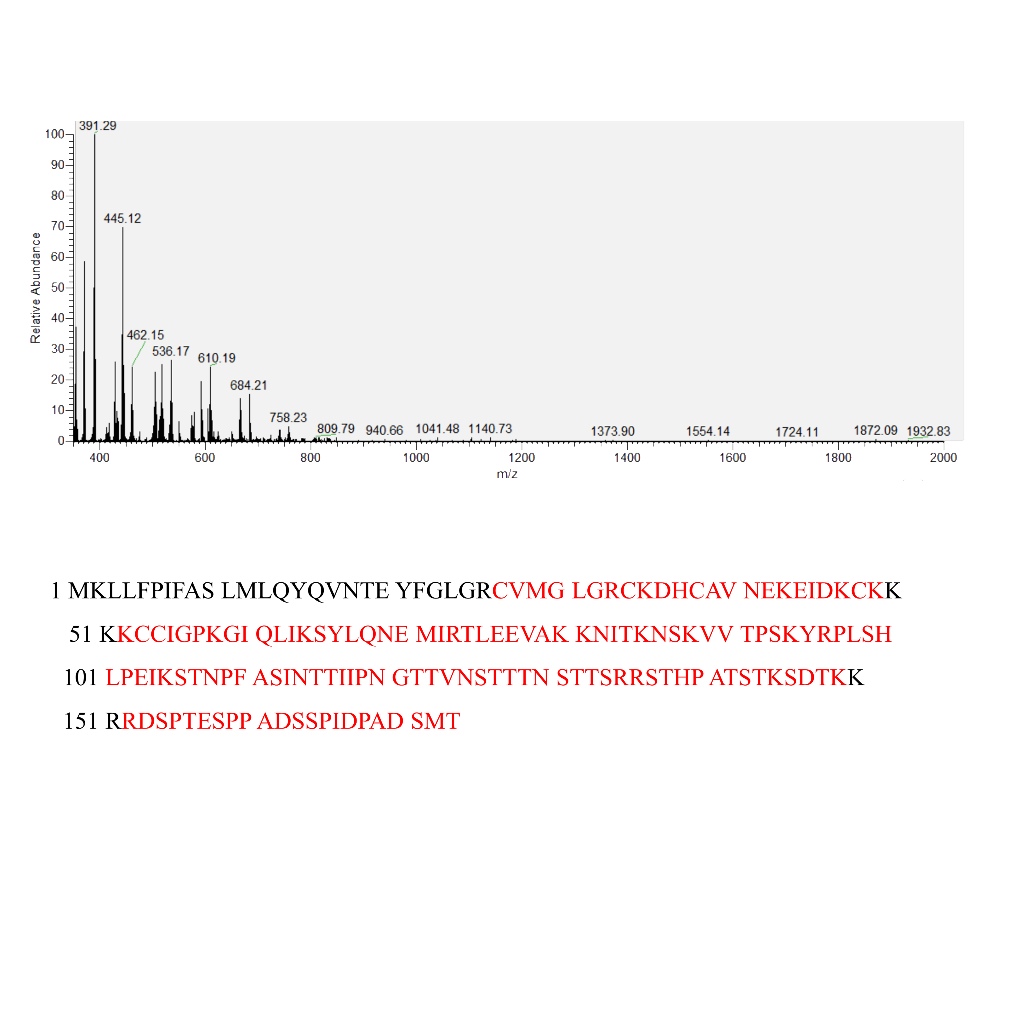

Supplement: Supplementary file 3 [file Image_2.TIF]
